# Personality Traits and Empathy in Relation to Attitudes About Communication Between Medical Students and Patients

**DOI:** 10.3390/bs15050683

**Published:** 2025-05-16

**Authors:** Lorena Mihaela Grebenisan, Andreea Sima-Comaniciu, Emese Erika Lukacs, Aurel Nireștean, Gabriela Elena Strete, Horia Marchean, Bianca Larisa Abalasei, Andrei Cotruș, Alex-Claudiu Boacă, Ileana Marinescu, Adriana Mihai

**Affiliations:** 1 Department of Psychiatry, George Emil Palade University of Medicine, Pharmacy, Sciences, and Technology of Targu Mures, 540139 Targu Mures, Romania; lorena-mihaela.muntean@umfst.ro (L.M.G.);; 2 Mures County Hospital, Psychiatry Clinic No 2, 540136 Targu Mures, Romania; 3 Department of Psychology, Dimitrie Cantemir University from Targu Mures, 540139 Targu Mures, Romania; 4 Department of Psychiatry, University of Medicine and Pharmacy of Craiova, 200349 Craiova, Romania

**Keywords:** personality, empathy, medical students, attitudes towards learning communication skills

## Abstract

The medical profession requires continuous knowledge acquisition, effective communication skills, an appropriate level of empathy, and a personality profile that can support high-quality patient care. (1) The purpose of this study was to research whether there are associations or correlations between personality dimensions, empathy, and the attitudes of medical students regarding the learning of communication skills. (2) We conducted a pilot study with 267 first- and sixth-year medical students from the George Emil Palade University of Medicine, Pharmacy, Sciences, and Technology of Targu Mures as the subjects. The students were evaluated using the DECAS personality inventory, the Romanian communication skills ability scale, and Toronto empathy scale. (3) Our results showed that regarding the level of empathy (*p* = 0.09) and positive attitudes related to communication skills (*p* = 0.52), there were no statistically significant differences between first- and sixth-year medical students. On the other hand, in the case of negative attitudes, it was observed that there was a statistical significance (*p* = 0.0003). It was also observed that there was a positive association between agreeableness and empathy (OR = 6.12, *p* < 0.0001) and a negative association between emotional stability and empathy (OR = 0.45, *p* = 0.01). Correlations were also found between positive attitudes related to communication skills with patients and the personality dimensions of conscientiousness (r = 0.21, *p* = 0.0004) and agreeableness (r = 0.15, *p* = 0.01), as well as between negative attitudes related to communication skills with patients and the agreeableness dimension (r = −0.23, *p* = 0.0001) and emotional stability dimension (r = −0.13, *p* = 0.02). Furthermore, the two proposed models confirmed the influence that personality and empathy have on attitudes towards learning communication skills. (4) The findings of this study show that both the personality structure and the empathy of a student are linked to their attitudes about communication.

## 1. Introduction

A doctor should demonstrate professionalism, as it is an integral part of good medical practice, requiring up-to-date knowledge, technical skills, and communication skills, as well as values, empathy, and ethical competencies (Kirk, 2007; Dopelt et al., 2022).

Along with the traits mentioned above, personality plays a particularly important role (Scheepers et al., 2014). Thus, the personality structure, assessed dimensionally according to the big five model (also called the five-factor model (FFM)), influences interpersonal relationships and implicitly effective communication with patients (Sabzi & Yousefi, 2013). This is a multidimensional concept that integrates biological, psychological, biographical, and spiritual elements (Thuy, 2020). The FFM evaluates personality according to the following dimensions: openness, conscientiousness, extraversion, agreeableness, and emotional stability (Widiger & Crego, 2019).

Doctor–patient communication is the cornerstone of the therapeutic relationship (Sharkiya, 2023). It improves patient compliance, increases the quality of medical care, decreases potential medical errors, and increases patient satisfaction with their medical care (Kwame & Petrucka, 2021). Over time, studies have shown that students’ communication skills improve after taking specific courses on doctor–patient communication (Harlak et al., 2008).

Several theories are described in the literature that can be applied to attitudes toward learning communication skills. Albert Bandura’s theory, which indicates that individuals learn behavior through observation and imitation, shows that attitudes toward learning communication skills can be influenced by surrounding examples, such as teacher behavior, and it has also been demonstrated that students who are praised and rewarded can have positive attitudes about the possibility of learning communication skills (Horsburgh & Ippolito, 2018; Bandura, 1977, 2012).

It has been observed that women have more positive attitudes than men regarding the importance of communication skills (Koponen et al., 2012), and that students’ communication attitudes influence the way in which the doctor–patient communication course is completed, the time required to acquire the skills, and the way in which the student will put these skills into practice after graduation (Givron & Desseilles, 2021).

In general terms, empathy can be defined as the ability of a person to be able to transpose, understand, and feel what another person experiences (Mark, 2023). It is also well known that interpersonal relationships are highly dependent on understanding, sharing, and involvement, which are crucial for coexistence in society (Depow et al., 2021).

The therapeutic relationship between a doctor and their patient is crucial in the medical field, which is why empathy is essential (Derksen et al., 2013). A doctor’s ability to understand their patient’s emotions, feelings, and situation contributes to the development of a therapeutic relationship that will increase the patient’s confidence in the physician and encourage them to disclose relevant information for therapeutic conduct (Andersen et al., 2020). In addition, a relationship based on trust will increase therapeutic adherence. In this way, it will also increase the quality of the medical care, because the effort is bidirectional, both from the doctor and the patient (Eby, 2018). The neurobiological model of empathy is associated with neuroscience and biological psychiatry, with the preservation of connective capacities between neural systems, but also with the preservation of the neurotransmission constant. A possible level of genetic vulnerability to decreased or distorted empathy is debated. The good functionality of neurobiological support is associated with the neurotransmission constant, and dysfunction is associated with a decrease in the production of dopamine or serotonin by altering the genes that control the levels of these neurotransmitters (Stovicek et al., 2020; Marinescu et al., 2017; Dehelean et al., 2019).

Studies from the literature regarding empathy show that women are more patient-centered, and their doctor–patient communication is superior compared to men (Roter et al., 2002). It has also been demonstrated that empathy levels decrease as a student approaches graduation, with a slight recovery observed in the last year of college (Baig et al., 2023).

In addition, several theories can be applied to attitudes toward learning communication skills. Thus, an increased level of empathy in the theory of planned behavior can lead a student to consider that communication is not only a skill, but also a tool for understanding those around them, leading to a positive attitude (Burns et al., 2017). The theory of planned behavior is based on the fact that behavior is determined by the intention to perform the respective behavior and shaped by personal beliefs, perceived control, and social norms (Ajzen, 1991). Moreover, according to Deci and Ryan’s theory extrapolated to communication skills, empathetic students stimulate intrinsic motivation and psychological needs such as autonomy, competence, and relatedness to develop communication skills, in addition to viewing communication as a necessary tool in building and maintaining a therapeutic relationship (Neufeld & Malin, 2024; Ryan & Deci, 2014).

From a dimensional perspective of personality and empathy level, it has been highlighted that the dimensions of extraversion, openness, and agreeableness are associated with the level of empathy (Bętkowska-Korpała et al., 2021). Song and Shi demonstrated that even the dimension of conscientiousness is associated with the level of empathy in medical students (Song & Shi, 2017).

Regarding personality from a dimensional point of view, it is known that individuals with increased openness are more willing to engage in learning communication skills, want to experiment with new methods of communication, and are interested in communicating more effectively (Karadağ & Kaya, 2017). Individuals whose conscientiousness is higher are more actively involved in learning communication skills (Hassan et al., 2019). Extroverts feel comfortable socializing, which is why they have a positive inclination and attitude towards learning communication skills (Karadağ & Kaya, 2017). The agreeableness dimension actually encompasses empathy, cooperation, and the desire to establish trust-based relationships with those around them, which is why individuals with a high level of agreeableness are more motivated to learn how to communicate with patients (Giuliani dos Santos Franco et al., 2020). Students with low values for the emotional stability dimension experience more negative feelings about those around them; they are less motivated to learn communication skills due to the fear that they might make mistakes or be judged (Molinuevo & Torrubia, 2013; Iwanow et al., 2021; Svensberg et al., 2017; Holmes et al., 2020; Giuliani dos Santos Franco et al., 2020).

On the basis of previous studies, we consider it important to know the particularities of the personalities of students, as well as their empathy level and their attitudes regarding the learning of communication skills, in order to develop academic programs that are structured according to their needs. Medical students’ attitudes toward the learning of communication skills are influenced by higher levels of empathy and personality traits such as the conscientiousness, agreeableness, and emotional stability dimensions from the big five approach.

## 2. Methodology

### 2.1. Design of the Study

The present study was an observational–transversal pilot-type study that involved medical students in their 1st and 6th years at the G.E. Palade UMPhST of Targu Mures as the subjects. The research was approved by the Ethics Committee of the G.E. Palade UMPhST of Targu Mures, decision no. 2937/18.03.2024, and it was carried out in the period from 19 March 2024 to 5 May 2024.

### 2.2. Data Collection Process

Data collection took place between 19 March and 5 May 2024. During this period, students were invited to participate on a voluntary basis by completing a set of questionnaires related to their personality traits, empathy, and attitudes toward learning communication skills. Participation was anonymous and did not influence academic standing. Social media was used to distribute the surveys online in medical groups to students from the G.E. Palade UMPhST of Targu Mures. In the 2023–2024 academic year, approximately 220 students were enrolled in the first year and 260 students were enrolled in the sixth year of the Romanian-language medical program. These figures reflect the enrollment in the Romanian teaching line, and are comparable to the numbers recorded in the 2022–2023 academic year (Rector’s annual report regarding the Status of George Emil Palade University of Medicine, Pharmacy, Science, and Technology of Targu, Mures, 2023, available online: https://www.umfst.ro/fileadmin/documente_oficiale/2024/Raportul_anual_al_rectorului_privind_starea_UMFST_GEP_Tg%20Mures_2023_26.03.2024_old.pdf accessed on 15 January 2025). Subjects from the 1st and 6th years were chosen because, in 2nd year, students follow the course “Doctor–patient communication”, during which they develop communication skills and an understanding of the importance of proper communication between the doctor and patient; this can change their attitude related to communication. We also took into account the fact that students who are in their 6th year already have practical experience due to patient contact. The participants completed the following scales: the DECAS personality inventory (DECAS), the Romanian communication skills ability scale (CSAS-RO), and the Romanian version of the Toronto empathy questionnaire (TEQ). In addition to these scales, this study included the following parameters: age, gender, and year of study.

In this study, we included 267 students out of a total of 285 participants. A total of 18 students were excluded because they did not meet the eligibility criteria for participation; specifically, the internal validation scales of the DECAS showed distortions in their responses. The sampling was carried out according to the year of study, taking into account the fact that those in the 6th year followed the “doctor–patient communication” course. Thus, one group included 140 students from the 1st year and the other group included 127 subjects from the 6th year.

The inclusion criteria were as follows: students enrolled in the Faculty of Medicine’s first and sixth years of the G.E. Palade UMPhST of Targu Mures; students who gave their consent to be included in the study; and students who fully completed all the scales.

The exclusion criteria were as follows: students who provided distorted responses when comparing the DECAS’s internal validation scales and students from other study programs.

### 2.3. Data Collection Tools

#### 2.3.1. The DECAS Personality Inventory (DECAS)

DECAS was created by Sava and colleagues to evaluate personality according to the big five model. The scale has been validated for the Romanian population. Cronbach’s alpha coefficient for each dimension was calculated as follows: for openness, it was 0.71; for extraversion, it was 0.75; for conscientiousness, it was 0.7; for agreeableness, it was 0.71; and for emotional stability, it was 0.75. The scale is composed of 97 assertions that evaluate personality from a dimensional perspective. Individuals with an increased openness dimension are concerned with art and culture, are curious, and have high standards, whereas those at the opposite pole are traditionalists, respect the rules, and do not experience new things. Extroverted individuals are sociable, optimistic, enthusiastic, and active, while introverted individuals are passive, do not take initiative, and prefer to work alone. People with high values for the conscientiousness dimension are ambitious, organized, persistent, and have a high capacity for self-control; at the opposite pole are people with low values of conscientiousness, who cannot be relied upon, are relaxed, and do not respect deadlines. Agreeable people are empathetic and oriented towards the needs of those around them, showing cooperation and altruism, while individuals with low values for the agreeableness dimension are competitive and self-centered. Individuals who have high values for the emotional stability dimension are relaxed, easily handle crisis situations, and cope with stress easily, while those on the opposite pole are prone to experiencing anxiety and depression. The tool also includes three validation scales to avoid distorted answers: social desirability (SD), which measures the tendency of subjects to answer questions in a way that puts themselves in a good light; random answers (RD), which measures the tendency of subjects to give random answers; and approving answers (AP), which measures the tendency of subjects to provide either “true” or “false” responses. The scoring system for the scale is interpreted in the following way: values from 20 to 35 are very low, values from 35 to 45 are low, values from 45 to 55 are average, values from 55 to 65 are high, and values of 65–80 indicate very high values for the dimensions (Sava, 2008). The designated value for conducting statistical tests and interpretation was 45 (Muntean et al., 2022).

#### 2.3.2. Toronto Empathy Questionnaire (TEQ)

The TEQ was developed by Spreng et al. This questionnaire consists of 16 items, which are scored on a Likert scale with 5 points as follows: 0 = never, 1 = rarely, 2 = sometimes, 3 = often, and 4 = always. The responses are summed to calculate a total empathy score. For items 2, 4, 7, 10, 11, 12, 14, and 15, the score is reversed, while for items 1, 3, 5, 6, 8, 9, 13, and 16, the score is summed. The obtained score varies between 0 and 64. High scores indicate more empathy. Scores below 45 indicate low levels of empathy. The questionnaire was validated on the Romanian population with a Cronbach’s alpha coefficient = 0.72, which demonstrates the excellent reliability and internal consistency of the TEQ (Ursoniu et al., 2021).

#### 2.3.3. Communication Skills Ability Scale (CSAS-RO)

The CSAS-RO was created by Rees and colleagues in 2002. This questionnaire has 26 items framed within two subscales: the positive attitude scale (PAS), which includes items 4, 5, 7, 9, 10, 12, 14, 16, 18, 21, 22, 23, and 25, and the negative attitude scale (NAS), which includes items 2, 3, 6, 8, 11, 13, 15, 17, 19, 20, 24, and 26. The score for the PAS is obtained from the sum of the 13 items, with the mention that the value of item 22 must be reversed. The NAS score is calculated by summing the 12 items and reversing the score of item 1. Both scales have values between 13 and 65, and higher scores mean stronger positive or negative attitudes (Yakhforoshha et al., 2018). The scale was validated on the Romanian population, with Cronbach’s alpha coefficient being 0.89 (Steriu et al., 2022). To carry out the statistical analysis, a value of 52 was set as the standard for the PAS, representing the average of the values obtained by the students included in the study. A value of 24 was set as the reference value in the case of the NAS, representing the average of the values obtained by the students, in order to perform the statistical analysis.

### 2.4. Statistical Analysis

The database was created using the Microsoft Office Excel 2021 software. Graph Pad Prism 8 was used to perform the statistical analysis. α was set at 0.05 to interpret the results, with a confidence interval (CI) of 95%. Utilizing the Kolmogorov–Smirnov test, the data distribution’s normality was examined. The Mann–Whitney test for non-parametric data was used to compare the level of empathy and the communication-related attitudes of students according to the year of study. The chi^2^ test was used to perform a contingency table analysis to test the associations between personality dimensions and the level of empathy. The Spearman test was used to test the correlations between positive and negative attitudes related to communication skills and personality dimensions. In order to test the influence of personality and empathy on positive and negative attitudes toward learning communication skills, multiple linear regression was used.

## 3. Results

This study included 267 subjects, of whom 199 (74.53%) were women and 68 (25.47%) were men. The initial number of subjects that finalized the scales was 285, and 18 participants were excluded because they did not meet the eligibility criteria for participation. Of the subjects included in the study, 140 (52.43%) were in their first year and 127 (47.57%) were in their sixth year of study. The respondents fell within the age range of 18–42 years. Regarding the environment of origin, 189 (70.78%) students came from an urban area and 78 (29.22%) came from a rural area (Table 1).

Regarding the level of empathy, there were no statistically significant differences between students in their first year (48.73 ± 7.30) and those in their sixth year (50.32 ± 6.86, *p* = 0.09). Regarding positive attitudes related to communication skills, there were also no statistically significant differences between the students in their first year (53.17 ± 6.92) and those in their sixth year (52.76 ± 6.91, *p* = 0.52). On the other hand, in the case of negative attitudes, it was observed that there was a statistically significant difference between first-year students (25.41 ± 5.19) and sixth-year ones (23.13 ± 4.22, *p* = 0.0003). The abovementioned information can be found in Table 2.

Table 3 shows that there was a positive association between a negative attitude related to communication skills and the year of study (OR = 1.96, *p* = 0.006). No correlation between positive attitudes related to student–patient communication and the study year was found (OR = 1.51, *p* = 0.1). There were also no statistically significant associations between the level of empathy and the year of study (OR = 0.69, *p* = 0.2).

Regarding personality dimensions, it was observed that there were positive correlations between the conscientiousness (r = 0.21, *p* = 0.0004) and agreeableness (r = 0.15, *p* = 0.01) dimensions and positive attitudes towards communication skills. No statistically significant differences were highlighted between the openness (r = 0.05, *p* = 0.41), extraversion (r = 0.11, *p* = 0.06), or emotional stability (r = −0.007, *p* = 0.90) dimensions and positive attitudes towards communication skills.

In the case of negative attitudes towards communication skills, it was observed that there was a negative correlation with the dimensions of agreeableness (r = −0.23, *p* = 0.0001) and emotional stability (r = −0.13, *p* = 0.02). No correlations were found between negative attitudes towards communication skills and the dimensions of openness (r = 0.03, *p* = 0.61), extroversion (r = −0.03, *p* = 0.61), or conscientiousness (r = −0.03, *p* = 0.59). All of the data mentioned above are presented in Table 4.

Regarding personality dimensions and the level of empathy in Table 5, we found that the agreeableness dimension was positively associated with the empathy level (OR = 6.12, *p* < 0.0001) as well as the dimension of emotional stability (OR = 0.45, *p* = 0.01). In the case of the openness (OR = 1.44, *p* = 0.33), extraversion (OR = 1.57, *p* = 0.11), and conscientiousness (OR = 1.75, *p* = 0.08) dimensions, it was observed that there were no statistically significant associations with the level of empathy

Multiple linear regression demonstrated that the conscientiousness dimension, together with empathy, influenced positive attitudes related to learning communication skills. Thus, students who have higher levels of the conscientiousness dimension will have positive attitudes towards communication (β = 0.116, *p* = 0.0016). In addition, the level of empathy (β = 0.337, *p* < 0.0001) was an important predictor, confirming that increased levels of empathy are associated with a more favorable positive attitude toward learning communication skills. Regarding the dimensions of openness (β = −0.032, *p* = 0.43), extraversion (β = 0.048, *p* = 0.22), agreeableness (β = 0.071, *p* = 0.12), and emotional stability (β = −0.007, *p* = 0.88), no dependence relationships with positive attitudes towards communication skills were highlighted. The model explained 19.4% of the variance, indicating a moderate size effect. All of the data mentioned above are presented in Table 6 and Table 7.

The model explained approximately 19.57% of the variance in the negative attitudes toward learning communication skills, indicating a moderate effect size. Regarding personality dimensions, it was highlighted that increased values of the agreeableness dimension (β = −0.077, *p* = 0.01) resulted in a low level of negative attitudes related to communication skills. Regarding the emotional stability dimension (β = −0.086, *p* = 0.005), it was highlighted that students with high values of this trait tend to have a less negative attitude toward communication skills. It was also highlighted that more empathetic students (β = −0.246, *p* < 0.0001) have a less negative attitude about communication skills. No predictions were found regarding the dimensions of openness (β = 0.041, *p* = 0.15), extraversion (β = 0.013, *p* = 0.62), or conscientiousness (β = 0.007, *p* = 0.77) in relation to negative attitudes about learning communication skills. The multiple linear regression results that predict a negative attitude toward learning communication skills are found in Table 8 and Table 9.

## 4. Discussion

According to the literature, an empathic doctor with appropriate communication skills can lead to a better therapeutic relationship, and the quality of medical treatment can be improved by increasing therapeutic compliance (Arshad et al., 2024; Kim et al., 2004). Medical errors can be minimized through proper communication, as detailed anamnesis and heteroanamnesis can provide useful information for case management (Tiwary et al., 2019).

Considering the literature and the fact that, to our knowledge, the relationship in Romania between personality, empathy, and attitudes related to communication with patients has not been investigated, the purpose of the current study was to investigate whether medical students’ attitudes towards learning communication skills are influenced by higher levels of empathy and personality traits from the big five approach.

The results obtained indicate that there are no statistically significant differences or associations in the positive beliefs between first-year and sixth-year students. This can be explained by the fact that both student groups understood the necessity for effective communication between a doctor and a patient and they believed that it is necessary to learn and be motivated, attentive, and willing to make the necessary effort in this direction (Taveira-Gomes et al., 2016). In the case of negative beliefs, there is a statistically significant difference between first-year students who have stronger negative beliefs compared to those in their sixth year regarding the learning of doctor–patient communication skills. A statistically significant association between negative beliefs and the year of study was also observed. The experience gained by sixth-year students over time and the system structure in which the doctors work helps students understand the need to communicate effectively with patients, resulting in fewer negative beliefs related to learning and increased confidence and openness towards doctor–patient communication (Keshavarzi et al., 2022; King & Hoppe, 2013; Street et al., 2003). The study conducted by Alotaibi and Alsaeedi demonstrated that both fifth-year students and elderly individuals have positive attitudes towards communication skills (Alotaibi & Alsaeedi, 2016). According to research by Shankar et al., lower values were obtained in the negative attitude sub-scale for respondents aged over 30 years and medical students in their fourth year (Shankar et al., 2013). Ruiz-Moral et al. conducted another study and found that the level of positive attitude scale related to students’ communication skills decreased over the years, while the negative attitude scale values increased (Ruiz-Moral et al., 2021).

Empathy is essential in the medical profession to understand a patient’s concerns, experiences, and the expectations they have for the doctor (Moudatsou et al., 2020). Regarding the level of empathy, no statistically significant differences between the two groups of students were obtained. No associations were found between the empathy level and the year of study. There were also no statistically significant differences in the level of empathy between year I and year VI. This study’s results have been confirmed by other studies, such as that by Quince et al. (Quince et al., 2016), but there have also been studies indicating significant differences based on the medical student’s year of study (Hizomi Arani et al., 2021). The research conducted by Magalhães et al. revealed a higher level of empathy among students in their final years (Magalhães et al., 2011). Instead, Baig et al. demonstrated that, throughout medical education, the level of empathy decreases, and there is a slight recovery in the last year (Baig et al., 2023). The decline in empathy levels can be explained by the constant and increased stress that doctors are exposed to, the increased number of patients they need to treat, limited resources, and the lack of a mentor (Akgün et al., 2020).

When it comes to personality dimensions, this study revealed a positive correlation between conscientiousness, agreeableness, and positive beliefs related to improving communication skills.

In terms of personality dimensions, this study revealed that there was a positive association between conscientiousness, agreeableness, and positive beliefs related to learning communication skills. The conscientiousness dimension is characterized by organization, meticulousness, perseverance, and ambition, which is why students with higher conscientiousness values are more likely to have a positive attitude towards the possibility of learning new communication skills than those with lower values (Roberts et al., 2014). Regarding the conscientiousness dimension, Schneider et al. showed that doctors with higher values of conscientiousness can better handle uncertain situations and communicate more effectively (Schneider et al., 2014). The agreeableness dimension, which allows an individual to be attentive to the needs of others, empathic, honest, modest, and altruistic (Sheese & Graziano, 2004), is also correlated positively with positive beliefs related to communication with patients. It has been shown in previous research that the agreeableness, openness, and extraversion dimensions influence the communication between a doctor and their patient, representing an important factor in patient satisfaction towards their doctor (Liu et al., 2022; Shankar et al., 2013). Our research indicates that the agreeableness and emotional stability dimensions are negatively correlated when it comes to students’ negative beliefs about communication with a patient. This can be explained by the fact that a student who is oriented towards their own needs can sometimes be hostile and can experience an increased level of anxiety, impulsivity, and vulnerability; hence, they do not consider it necessary to improve their communication skills (Giuliani dos Santos Franco et al., 2020).

The empathy level and personality dimensions are closely connected, as personality can have a significant impact on empathic behavior (Yasien & Almuzaini, 2022). Our research revealed that there is a positive association between the agreeableness dimension and empathy, and a negative association between the emotional stability dimension and empathy. A higher level of empathy and increased agreeableness can cause a higher level of stress, given that empathic doctors are more likely to feel the unpleasant emotions of patients (Bętkowska-Korpała et al., 2021; Magalhães et al., 2012). It was also highlighted that the emotional stability dimension is negatively correlated with the level of empathy; thus, doctors who show a higher level of stress, who cannot control negative emotions very well, and who are more vulnerable show a lower level of empathy (Chen, 2023). Considering the information mentioned above, it is crucial for doctors to maintain a balance between workplace involvement and patient care to prevent compassion fatigue (Garnett et al., 2023).

Regarding the relationship between attitudes about learning communication skills, empathy, and personality, the two proposed models highlighted the following. In the case of positive attitudes, there is a predictive association with the dimensions of conscientiousness and empathy. Students with higher levels of conscientiousness and empathy demonstrate a greater willingness and have positive attitudes regarding the possibility of learning communication skills. In the case of negative attitudes, the proposed model highlighted the fact that increased values of the agreeableness and emotional stability dimensions, as well as that of empathy, were associated with less negative attitudes related to learning communication skills. The two models had only a moderate impact, which indicates that they only partially explain the influence of personality and empathy on the attitudes toward learning communication skills. This suggests that additional factors contribute to learning. Variables such as intrinsic motivation, prior educational or interpersonal experiences, teaching methods, professional role models, and social and cultural values may be responsible for students’ openness and desire to learn communication skills (Gamage et al., 2021; Iversen et al., 2021; Douglas et al., 2021; Theron et al., 2025).

Since a good doctor is formed during their time as a student, it is important that students receive the necessary training to develop their communication skills during their time as both a student and a doctor (Deveugele et al., 2005). An individual’s attitude related to communication with patients depends on both their personality and level of empathy (Grilo et al., 2023). According to the literature, an enhanced level of empathy and agreeableness will facilitate a student’s ease of involvement in communication with patients (Song & Shi, 2017). In the case of increased values of empathy and openness, students will actively engage in and seek solutions to better understand the emotional needs of a patient. However, in the case of a low level of empathy and emotional stability, communication will be difficult because the student cannot understand the patients’ feelings and the attitude related to communication will be negative (Zohoorian et al., 2022). In the case of low empathy and high extroversion, the student will tend to dominate the conversation, not understand the patient’s emotions, and have a negative attitude towards communicating with patients (Wan et al., 2019).

Our findings can be better understood through the lens of several established behavior change theories. Ajzen’s theory suggests that human behavior is determined by the intention to perform the respective behavior and shaped by personal beliefs, perceived control, and social norms (Ajzen, 1991). Thus, students’ positive attitudes toward learning communication skills and their perceptions of their ability to learn them may influence their behavior in engaging in patient communication learning programs. Also, social norms represented by social pressure and expectations of others may have a significant impact on the choice to engage in these activities along with the level of empathy. According to Deci and Ryan’s Theory (Deci & Ryan, 2000), applied to the results obtained by us, we can assume that students with a higher level of empathy are more oriented towards learning effective communication skills, considering them, in addition to a practical skill, a binder in professional and interpersonal relationships. Given Bandura’s Social Cognitive Theory that behavior is learned through observation and imitation (Bandura, 1986, 1977, 2012), future research may address educational contexts, teaching methods, and professional role models to assess the development of attitudes toward learning communication skills. In particular, exposing students to teachers or mentors who effectively demonstrate communication skills could significantly influence the development of motivation and self-efficacy in this area.

In conclusion, attitudes towards learning communication skills are influenced by an individual’s level of empathy, their personality, and external factors. An empathetic student will more easily understand the importance of proper communication with patients and will have a positive attitude toward the possibility of learning communication techniques, unlike a student who has a lower level of empathy (Babaii et al., 2021). More empathetic students are willing to learn and do not become defensive when obstacles arise. Empathy improves communication, but at the same time, it also facilitates a positive attitude towards learning communication skills (Harlak et al., 2008).

By understanding the attitudes related to learning communication skills among medical students, as well as how personality and empathy influence them, universities of medicine can take necessary measures to introduce doctor–patient communication courses into the university curriculum (Shankar et al., 2013). Education in communication and empathy can decrease stress, anxiety, and exhaustion at work, which can lead to improved medical care quality (Bętkowska-Korpała et al., 2022). Along with the structure of personality and self-knowledge, the constant training of students and doctors is mandatory to ensure the best care for patients (Suciu et al., 2021).

## 5. Limitations

The first limitation is that this study was a pilot and only included students from one university, namely the G.E. Palade UMPhST of Targu Mures, which resulted in a relatively small sample. In the future, it may be beneficial to carry out a national study in which several medical universities in the country are included. Due to the fact that this was a pilot study, further research is needed to validate the assumptions generated. This study was based on observational–transversal research and did not follow the same students over time to observe whether their attitudes towards communication skills changed due to experience or their beliefs. Future longitudinal research would provide an interesting perspective on how empathy evolves over time. In addition, future research should take into account variables such as intrinsic motivation, interpersonal experiences, educational contexts, teaching methods, and professional role models in order to obtain a more comprehensive understanding of students’ attitudes toward learning communication skills. Also, another limitation may be represented by the lack of randomization due to the fact that the sample was selected through participants who wanted to participate in the research, which may generate a selection bias. Another limitation is represented by the fact that cognitive and affective empathy were not studied to understand which of the two influences attitudes related to communication skills more. Also, the level of empathy was measured through self-assessment questionnaires that may not accurately reflect empathic behavior in real contexts, given that responses may be influenced by the desire to conform to expectations or idealized perceptions of one’s own behavior. Furthermore, the fact that the research is based on self-assessment and responses may be biased since only the DECAS personality inventory has an internal scale to highlight possible distorted responses, which may represent another important limitation.

## 6. Conclusions

This study provides preliminary data on the relationships between personality, empathy, and student attitudes related to communication with patients. It was demonstrated that there is a positive association between the agreeableness dimension and empathy, as well as a negative association between the emotional stability dimension and empathy. It was also shown that there are correlations between positive and negative attitudes related to patient communication skills and the personality dimensions of conscientiousness, agreeableness, and emotional stability. According to the two proposed models, about 20% of the variation in attitudes related to learning communication skills is due to empathy and personality, while the remainder is due to internal and external factors. The validation of these results on a larger scale could be used to develop educational programs that offer counseling to students to deepen their self-knowledge and develop their personality through dimensions and motivations, which would improve communication with patients.

## Figures and Tables

**Table 1:** Demographic analysis.

Participant Characteristics	n = 267
Gender, n (%)	
Female	199 (74.53)
Male	68 (25.47)
Age range (M ± SD)	18–42 (22 ± 3.2)
Study year, n (%)	
1	140 (52.43)
6	127 (47.57)
Area of origin, n (%)	
Urban	189 (70.78)
Rural	78 (29.22)

Legend: M, mean; SD, standard deviation.

**Table 2:** Evaluation of the level of empathy and attitudes towards communication skills according to the year of study.

Variables	First Year (n = 140) M ± SD 95% CI	Sixth Year (n = 127) M ± SD 95% CI	*p* ^a^
Toronto scale	48.73 ± 7.30 (47.52–49.93)	50.32 ± 6.86 (49.12–51.51)	0.09
CSAS-RO scale			
PAS	53.17 ± 6.92 (52.02–54.31)	52.76 ± 6.91 (51.55–53.96)	0.52
NAS	25.41 ± 5.19 (24.54–26.28)	23.13 ± 4.22 (22.39–23.88)	0.0003

Legend: CI, confidence interval; M, mean; SD, standard deviation; ^a^ Mann–Whitney test; CSAS-RO, communication skills ability scale; PAS, positive attitude scale; NAS, negative attitude scale.

**Table 3:** Associations between the level of empathy, the attitudes towards communication skills, and the year of study.

Variables	Year of Study		
	OR	95% CI	*p* ^b^
Toronto scale	0.69	0.38–1.23	0.2
CSAS scale			
PAS	1.51	0.93–2.48	0.1
NAS	1.96	1.2–3.15	0.006

Legend: CI, confidence interval; OR, odds ratio; ^b^ chi^2^ test; CSAS-RO, communication skills ability scale; PAS, positive attitude scale; NAS, negative attitude scale.

**Table 4:** The Spearman correlations between personality dimensions and positive and negative attitudes towards communication skills.

DECAS	CSAS-RO SCALE					
	PAS			NAS		
	r	95% CI	*p*	r	95% CI	*p*
Openness	0.05	−0.07–0.17	0.41	0.03	−0.09–0.15	0.61
Extraversion	0.11	−0.01–0.23	0.06	−0.03	−0.15–0.09	0.61
Conscientiousness	0.21	0.09–0.33	0.0004	−0.03	−0.15–0.09	0.59
Agreeableness	0.15	0.02–0.26	0.01	−0.23	−0.34–−0.11	0.0001
Emotional Stability	−0.007	−0.13–0.11	0.90	−0.13	−0.25–−0.01	0.02

Legend: r, Spearman’s correlation coefficient; CI, confidence interval; CSAS-RO, communication skills ability scale; PAS, positive attitude scale; NAS, negative attitude scale; DECAS, the DECAS personality inventory.

**Table 5:** Associations between personality dimensions and empathy level.

DECAS	Empathy		
	OR	95% CI	*p* ^b^
Openness	1.44	0.68–3	0.33
Extraversion	1.57	0.89–2.72	0.11
Conscientiousness	1.75	0.91–3.31	0.08
Agreeableness	6.12	2.9–13.37	<0.0001
Emotional Stability	0.45	0.24–0.88	0.01

Legend: CI, confidence interval; OR, odds ratio; ^b^ chi^2^ test; DECAS, the DECAS personality inventory.

**Table 6:** Multiple linear regression results predicting positive attitudes toward learning communication skills.

Predictor	β	SE	t	95% CI	*p*
Intercept	26.05	4.949	5.265	16.31 to 35.80	<0.0001
Openness	−0.032	0.04	0.782	−0.111 to 0.048	0.43
Extraversion	0.048	0.04	1.206	−0.030 to 0.127	0.22
Conscientiousness	0.116	0.036	3.188	0.044 to 0.188	0.0016
Agreeableness	0.071	0.046	1.529	−0.020 to 0.162	0.12
Emotional Stability	−0.007	0.044	0.15	−0.092 to 0.079	0.88
Empathy	0.337	0.057	5.87	0.224 to 0.450	<0.0001

Legend: CI, confidence interval; SE, standard error; dependent variable: positive attitude toward learning communication skills; independent variables: openness, extraversion, conscientiousness, agreeableness, emotional stability, and empathy.

**Table 7:** Goodness-of-fit statistics for the regression model explaining positive attitudes toward learning communication skills.

Goodness-of-Fit Measure	Value	*p*
R^2^	0.1940	<0.0001
df	260	

Legend: df, degrees of freedom.

**Table 8:** Multiple linear regression results predicting negative attitudes toward learning communication skills.

Predictor	β	SE	t	95% CI	*p*
Intercept	41.61	3.495	11.90	34.72 to 48.49	<0.0001
Openness	0.041	0.028	1.44	0.015 to 0.097	0.15
Extraversion	0.013	0.028	0.49	−0.041 to 0.069	0.62
Conscientiousness	0.007	0.025	0.28	−0.043 to 0.057	0.77
Agreeableness	−0.077	0.032	−2.36	−0.141 to −0.012	0.01
Emotional Stability	−0.086	0.03	−2.80	−0.146 to −0.025	0.005
Empathy	−0.246	0.04	−6.09	−0.326 to −0.167	<0.0001

Legend: CI, confidence interval; SE, standard error; dependent variable: negative attitude toward learning communication skills; independent variables: openness, extraversion, conscientiousness, agreeableness, emotional stability, and empathy.

**Table 9:** Goodness-of-fit statistics for the regression model explaining negative attitudes toward learning communication skills.

Goodness-of-Fit Measure	Value	*p*
R^2^	0.1957	<0.0001
df	260	

Legend: df, degrees of freedom.

## Data Availability

The original contributions presented in this study are included in the article. Further inquiries can be directed to the corresponding authors.

## Abbreviations

The following abbreviations are used in this manuscript:

FFM	five-factor model
DECAS	the DECAS personality inventory
CSAS-RO	communication skills ability scale
TEQ	Toronto empathy questionnaire
PAS	positive attitude scale
NAS	negative attitude scale
OR	odds ratio
CI	confidence interval
df	degrees of freedom

## References

[B1-behavsci-15-00683] Ajzen I. (1991). The theory of planned behavior. Organizational Behavior and Human Decision Processes.

[B2-behavsci-15-00683] Akgün Ö., Akdeniz M., Kavukcu E., Avcı H. H. (2020). Medical students’empathy level differences by medical year, gender, and specialty interest in Akdeniz university. Journal of Medical Education and Curricular Development.

[B3-behavsci-15-00683] Alotaibi F. S., Alsaeedi A. (2016). Attitudes of medical students toward communication skills learning in Western Saudi Arabia. Saudi Medical Journal.

[B4-behavsci-15-00683] Andersen F. A., Johansen A. S. B., Søndergaard J., Andersen C. M., Hvidt E. A. (2020). Revisiting the trajectory of medical students’ empathy, and impact of gender, specialty preferences and nationality: A systematic review. BMC Medical Education.

[B5-behavsci-15-00683] Arshad M., Sriram S., Khan S., Gollapalli P. K., Albadrani M. (2024). Mediating role of physician’s empathy between physician’s communication and patient’s satisfaction. Journal of Family Medicine and Primary Care.

[B6-behavsci-15-00683] Babaii A., Mohammadi E., Sadooghiasl A. (2021). The meaning of the empathetic nurse-patient communication: A qualitative study. Journal of Patient Experience.

[B7-behavsci-15-00683] Baig K. S., Hayat M. K., Khan M. A. A., Humayun U., Ahmad Z., Khan M. A. (2023). empathy levels in medical students: A single center study. Cureus.

[B8-behavsci-15-00683] Bandura A. (1977). Self-efficacy: Toward a unifying theory of behavioral change. Psychological Review.

[B9-behavsci-15-00683] Bandura A. (1986). Social foundations of thought and action: A social cognitive theory.

[B10-behavsci-15-00683] Bandura A., Van Lange P. A. M., Kruglanski A. W., Higgins E. T. (2012). Social cognitive theory. Handbook of theories of social psychology.

[B11-behavsci-15-00683] Bętkowska-Korpała B., Epa R., Sikora-Zych K., Olszewska-Turek K., Pastuszak-Draxler A., Rajtar-Zembaty A., Starowicz-Filip A. (2021). Differences in personality related determinants of empathetic sensibility in female and male students of medicine. PLoS ONE.

[B12-behavsci-15-00683] Bętkowska-Korpała B., Pastuszak-Draxler A., Olszewska-Turek K., Epa R., Starowicz-Filip A. (2022). Personality characteristics of empathy profiles—Practical implications for education of medicine students. BMC Medical Education.

[B13-behavsci-15-00683] Burns M. E., Houser M. L., Farris K. L. (2017). Theory of planned behavior in the classroom: An examination of the instructor confirmation-interaction model. Higher Education.

[B14-behavsci-15-00683] Chen Y. N. (2023). The relationship between personality traits, emotional stability and mental health in art vocational and technical college students during epidemic prevention and control. Psychology Research and Behavior Management.

[B15-behavsci-15-00683] Deci E. L., Ryan R. M. (2000). The “what” and “why” of goal pursuits: Human needs and the self-determination of behavior. Psychological Inquiry.

[B16-behavsci-15-00683] Dehelean L., Marinescu I., Stovicek P. O., Andor M. (2019). Cardiovascular anomalies and evolutionary risk factors in schizophrenia—Multifactorial approach. Romanian Journal of Morphology and Embryology = Revue Roumaine de Morphologie et Embryologie.

[B17-behavsci-15-00683] Depow G. J., Francis Z., Inzlicht M. (2021). The experience of empathy in everyday life. Psychological Science.

[B18-behavsci-15-00683] Derksen F., Bensing J., Lagro-Janssen A. (2013). Effectiveness of empathy in general practice: A systematic review. The British Journal of General Practice: The Journal of the Royal College of General Practitioners.

[B19-behavsci-15-00683] Deveugele M., Derese A., De Maesschalck S., Willems S., Van Driel M., De Maeseneer J. (2005). Teaching communication skills to medical students, a challenge in the curriculum?. Patient Education and Counseling.

[B20-behavsci-15-00683] Dopelt K., Bachner Y. G., Urkin J., Yahav Z., Davidovitch N., Barach P. (2022). Perceptions of practicing physicians and members of the public on the attributes of a “Good Doctor”. Healthcare.

[B21-behavsci-15-00683] Douglas A. H., Acharya S. P., Allery L. A. (2021). Communication skills learning through role models in Nepal; what are medical students really learning? A qualitative study. BMC Medical Education.

[B22-behavsci-15-00683] Eby D. (2018). Empathy in general practice: Its meaning for patients and doctors. The British Journal of General Practice: The Journal of the Royal College of General Practitioners.

[B23-behavsci-15-00683] Gamage K. A. A., Dehideniya D. M. S. C. P. K., Ekanayake S. Y. (2021). The role of personal values in learning approaches and student achievements. Behavioral Sciences.

[B24-behavsci-15-00683] Garnett A., Hui L., Oleynikov C., Boamah S. (2023). Compassion fatigue in healthcare providers: A scoping review. BMC Health Services Research.

[B25-behavsci-15-00683] Giuliani dos Santos Franco C. A. G., Franco R. S., Cecilio-Fernandes D., Severo M., Ferreira M. A. (2020). The assessment of personality traits and its association with learning communication skills. Scientia Medica.

[B26-behavsci-15-00683] Givron H., Desseilles M. (2021). Longitudinal study: Impact of communication skills training and a traineeship on medical students’ attitudes toward communication skills. Patient Education and Counseling.

[B27-behavsci-15-00683] Grilo A. M., Vinagre G., Santos M. C. D., Martinho J. F., Gomes A. I. (2023). Attitudes toward patient-centred care, empathy, and assertiveness among students in rehabilitation areas: A longitudinal study. Healthcare.

[B28-behavsci-15-00683] Harlak H., Gemalmaz A., Gurel F. S., Dereboy C., Ertekin K. (2008). Communication skills training: Effects on attitudes toward communication skills and empathic tendency. Education for Health.

[B29-behavsci-15-00683] Hassan N., Sumardi N. A., Aziz R. A. (2019). The influence of personality traits on communication competence. International Journal of Academic Research in Business and Social Sciences.

[B30-behavsci-15-00683] Hizomi Arani R., Naji Z., Moradi A., Shariat S. V., Mirzamohamadi S., Salamati P. (2021). Comparison of empathy with patients between first-year and last-year medical students of Tehran University of Medical Sciences. BMC Medical Education.

[B31-behavsci-15-00683] Holmes K. S., Zuckerman J. D., Maculatis M. C., Friedman A. M., Lawrence E., Phillips D. P. (2020). Personality predictors of communication skills among orthopedic surgery residents. Journal of Surgical Education.

[B32-behavsci-15-00683] Horsburgh J., Ippolito K. (2018). A skill to be worked at: Using social learning theory to explore the process of learning from role models in clinical settings. BMC Medical Education.

[B33-behavsci-15-00683] Iversen E. D., Wolderslund M., Kofoed P. E., Gulbrandsen P., Poulsen H., Cold S., Ammentorp J. (2021). Communication skills training: A means to promote time-efficient patient-centered communication in clinical practice. Journal of Patient-Centered Research and Reviews.

[B34-behavsci-15-00683] Iwanow L., Jaworski M., Gotlib J., Panczyk M. (2021). A model of factors determining nurses’ attitudes towards learning communicative competences. International Journal of Environmental Research and Public Health.

[B35-behavsci-15-00683] Karadağ Ş., Kaya Ş. D. (2017). The effects of personality traits on willingness to communicate: A study on university students. MANAS Sosyal Araştırmalar Dergisi.

[B36-behavsci-15-00683] Keshavarzi M. H., Safaie S., Faghihi S. A. A., Zare S. (2022). Barriers of physician-patient relationships in professionalism: A qualitative study. Journal of Advances in Medical Education & Professionalism.

[B37-behavsci-15-00683] Kim S. S., Kaplowitz S., Johnston M. V. (2004). The effects of physician empathy on patient satisfaction and compliance. Evaluation & the Health Professions.

[B38-behavsci-15-00683] King A., Hoppe R. B. (2013). “Best practice” for patient-centered communication: A narrative review. Journal of Graduate Medical Education.

[B39-behavsci-15-00683] Kirk L. M. (2007). Professionalism in medicine: Definitions and considerations for teaching. Proceedings.

[B40-behavsci-15-00683] Koponen J., Pyörälä E., Isotalus P. (2012). Comparing three experiential learning methods and their effect on medical students’ attitudes to learning communication skills. Medical Teacher.

[B41-behavsci-15-00683] Kwame A., Petrucka P. M. (2021). A literature-based study of patient-centered care and communication in nurse-patient interactions: Barriers, facilitators, and the way forward. BMC Nursing.

[B42-behavsci-15-00683] Liu M., Cai J., Chen H., Shi L. (2022). Association of personality traits with life and work of medical students: An integrative review. International Journal of Environmental Research and Public Health.

[B43-behavsci-15-00683] Magalhães E., Costa P., Costa M. J. (2012). Empathy of medical students and personality: Evidence from the Five-Factor Model. Medical Teacher.

[B44-behavsci-15-00683] Magalhães E., Salgueira A. P., Costa P., Costa M. J. (2011). Empathy in senior year and first year medical students: A cross-sectional study. BMC Medical Education.

[B45-behavsci-15-00683] Marinescu I., Enătescu V. R., Ghelase Ş. M., Marinescu D. (2017). Neurobiological arguments for a pathogenic multifactorial disconnective model of cognitive disorders from Alzheimer’s disease in elderly people. Romanian Journal of Morphology and Embryology = Revue Roumaine de Morphologie et Embryologie.

[B46-behavsci-15-00683] Mark H. D., Friedman H. S., Markey C. H. (2023). Empathy. Encyclopedia of mental health.

[B47-behavsci-15-00683] Molinuevo B., Torrubia R. (2013). Does personality predict medical students’ attitudes to learning communication skills?. International Journal of Medical Education.

[B48-behavsci-15-00683] Moudatsou M., Stavropoulou A., Philalithis A., Koukouli S. (2020). The role of empathy in health and social care professionals. Healthcare.

[B49-behavsci-15-00683] Muntean L. M., Nireștean A., Sima-Comaniciu A., Mărușteri M., Zăgan C. A., Lukacs E. (2022). The relationship between personality, motivation and academic performance at medical students from Romania. International Journal of Environmental Research and Public Health.

[B50-behavsci-15-00683] Neufeld A., Malin G. (2024). Cultivating physician empathy: A person-centered study based in self-determination theory. Medical Education Online.

[B51-behavsci-15-00683] Quince T. A., Kinnersley P., Hales J., da Silva A., Moriarty H., Thiemann P., Hyde S., Brimicombe J., Wood D., Barclay M., Benson J. (2016). Empathy among undergraduate medical students: A multi-centre cross-sectional comparison of students beginning and approaching the end of their course. BMC Medical Education.

[B52-behavsci-15-00683] (2023). Rector’s annual report regarding the Status of George Emil Palade University of Medicine, Pharmacy, Science, and Technology of Targu, Mures.

[B53-behavsci-15-00683] Roberts B. W., Lejuez C., Krueger R. F., Richards J. M., Hill P. L. (2014). What is conscientiousness and how can it be assessed?. Developmental Psychology.

[B54-behavsci-15-00683] Roter D. L., Hall J. A., Aoki Y. (2002). Physician gender effects in medical communication: A meta-analytic review. JAMA.

[B55-behavsci-15-00683] Ruiz-Moral R., Monge Martin D., Garcia de Leonardo C., Denizon S., Cerro Pérez A., Caballero Martínez F. (2021). Medical students’ attitudes towards communication skills training: A longitudinal study with one cohort. GMS Journal for Medical Education.

[B56-behavsci-15-00683] Ryan R. M., Deci E., Michalos A. C. (2014). Self-Determination Theory. Encyclopedia of quality of life and well-being research.

[B57-behavsci-15-00683] Sabzi N., Yousefi F. (2013). The role of personality dimensions in students’ communication skills. Quarterly Social Psychology Research.

[B58-behavsci-15-00683] Sava F. (2008). Inventarul de personalitate DECAS.

[B59-behavsci-15-00683] Scheepers R. A., Lombarts K. M. J. M. H., van Aken M. A. G., Heineman M. J., Arah O. A. (2014). Personality traits affect teaching performance of attending physicians: Results of a multi-center observational study. PLoS ONE.

[B60-behavsci-15-00683] Schneider A., Wübken M., Linde K., Bühner M. (2014). Communicating and dealing with uncertainty in general practice: The association with neuroticism. PLoS ONE.

[B61-behavsci-15-00683] Shankar P., Dubey A., Balasubramanium R., Dwivedi N. (2013). Student attitude towards communication skills learning in a Caribbean medical school. The Australasian Medical Journal.

[B62-behavsci-15-00683] Sharkiya S. H. (2023). Quality communication can improve patient-centred health outcomes among older patients: A rapid review. BMC Health Services Research.

[B63-behavsci-15-00683] Sheese B. E., Graziano W. G., Spielberger C. D. (2004). Agreeableness. Encyclopedia of applied psychology.

[B64-behavsci-15-00683] Song Y., Shi M. (2017). Associations between empathy and big five personality traits among Chinese undergraduate medical students. PLoS ONE.

[B65-behavsci-15-00683] Steriu A., Bratu E. C., Cioran N. V., Brînduşe L. A. (2022). Validation of the Romanian communication skills ability scale—CSAS-RO—A tool for medical schools. Journal of Medical Education and Curricular Development.

[B66-behavsci-15-00683] Stovicek P. O., Friedmann C., Marinescu D., Văduva I. A., Bondari S., Trifu S. C., Marinescu I. (2020). Mild TBI in the elderly—Risk factor for rapid cognitive impairment in Alzheimer’s disease. Romanian Journal of Morphology and Embryology = Revue Roumaine de Morphologie et Embryologie.

[B67-behavsci-15-00683] Street R. L., Krupat E., Bell R. A., Kravitz R. L., Haidet P. (2003). Beliefs about control in the physician-patient relationship: Effect on communication in medical encounters. Journal of General Internal Medicine.

[B68-behavsci-15-00683] Suciu N., Meliț L. E., Mărginean C. O. (2021). A holistic approach of personality traits in medical students: An integrative review. International Journal of Environmental Research and Public Health.

[B69-behavsci-15-00683] Svensberg K., Brandlistuen R. E., Björnsdottir I., Sporrong S. K. (2017). Factors associated with pharmacy students’ attitudes towards learning communication skills—A study among Nordic pharmacy students. Research in Social and Administrative Pharmacy.

[B70-behavsci-15-00683] Taveira-Gomes I., Mota-Cardoso R., Figueiredo-Braga M. (2016). Communication skills in medical students—An exploratory study before and after clerkships. Porto Biomedical Journal.

[B71-behavsci-15-00683] Theron L., Shopo K. D., Ngami O. (2025). Exploring perceptions of communication among culturally diverse nurses in Saudi Arabia. Journal of Transcultural Nursing: Official Journal of the Transcultural Nursing Society.

[B72-behavsci-15-00683] Thuy N. T. (2020). Personality traits and their influences on communication skills: The case of khmer students in Vietnam. International Journal of Innovation.

[B73-behavsci-15-00683] Tiwary A., Rimal A., Paudyal B., Sigdel K. R., Basnyat B. (2019). Poor communication by health care professionals may lead to life-threatening complications: Examples from two case reports. Wellcome Open Research.

[B74-behavsci-15-00683] Ursoniu S., Serban C. L., Giurgi-Oncu C., Rivis I. A., Bucur A., Bredicean A.-C., Papava I. (2021). Validation of the Romanian version of the Toronto empathy questionnaire (TEQ) among undergraduate medical students. International Journal of Environmental Research and Public Health.

[B75-behavsci-15-00683] Wan Q., Jiang L., Zeng Y., Wu X. (2019). A big-five personality model-based study of empathy behaviors in clinical nurses. Nurse Education in Practice.

[B76-behavsci-15-00683] Widiger T. A., Crego C. (2019). The Five Factor Model of personality structure: An update. World Psychiatry: Official Journal of the World Psychiatric Association (WPA).

[B77-behavsci-15-00683] Yakhforoshha A., Shirazi M., Yousefzadeh N., Ghanbarnejad A., Cheraghi M., Mojtahedzadeh R., Mahmoodi-Bakhtiari B., Emami S. A. H. (2018). Psychometric properties of the communication skills attitude scale (CSAS) measure in a sample of Iranian medical students. Journal of Advances in Medical Education & Professionalism.

[B78-behavsci-15-00683] Yasien S., Almuzaini F. (2022). The relationship between empathy and personality traits in Saudi medical students. Journal of Education and Health Promotion.

[B79-behavsci-15-00683] Zohoorian Z., Zeraatpishe M., Khorrami N. (2022). Willingness to communicate, big five personality traits, and empathy: How are they related?. Canadian Journal of Educational and Social Studies.

